# The Self-Enforcing Starch–Gluten System—Strain–Dependent Effects of Yeast Metabolites on the Polymeric Matrix

**DOI:** 10.3390/polym13010030

**Published:** 2020-12-23

**Authors:** Thekla Alpers, Viviane Tauscher, Thomas Steglich, Thomas Becker, Mario Jekle

**Affiliations:** 1 Research Group Cereal Technology and Process Engineering, Institute of Brewing and Beverage Technology, Technical University of Munich, 85354 Freising, Germany; thekla.alpers@tum.de (T.A.); tb@tum.de (T.B.); 2 Dr. Oetker Technology Development Center, 19243 Wittenburg, Germany; viviane.tauscher@gmx.de (V.T.); thomas.steglich@oetker.com (T.S.)

**Keywords:** yeast fermentation, wheat dough, strain hardening properties, fundamental extensional rheology, lubricated squeezing flow, shear rheology, protein network analysis, CLSM

## Abstract

The rheological behaviour of dough during the breadmaking process is strongly affected by the accumulation of yeast metabolites in the dough matrix. The impact of metabolites in yeasted dough-like concentrations on the rheology of dough has not been characterised yet for process-relevant deformation types and strain rates, nor has the effect of metabolites on strain hardening behaviour of dough been analysed. We used fundamental shear and elongational rheometry to study the impact of fermentation on the dough microstructure and functionality. Evaluating the influence of the main metabolites, the strongest impact was found for the presence of expanding gas cells due to the accumulation of the yeast metabolite CO_2_, which was shown to have a destabilising impact on the surrounding dough matrix. Throughout the fermentation process, the polymeric and entangled gluten microstructure was found to be degraded (−37.6% average vessel length, +37.5% end point rate). These microstructural changes were successfully linked to the changing rheological behaviour towards a highly mobile polymer system. An accelerated strain hardening behaviour (+32.5% SHI for yeasted dough) was promoted by the pre-extension of the gluten strands within the lamella around the gas cells. Further, a strain rate dependency was shown, as a lower strain hardening index was observed for slow extension processes. Fast extension seemed to influence the disruption of sterically interacting fragments, leading to entanglements and hindered extensibility.

## 1. Introduction

The behaviour of wheat dough, considered as a non-Newtonian fluid, is strongly dependent on the type and strength of stress applied. The processability is thereby determined by the viscoelastic properties of wheat dough, resulting from different interactions among the major) and minor dough components [1,2]. Due to the heterogeneous network configuration and the complex rheological behaviour, the response of the dough system is likely to depend on the type and strength of stress applied to the system. Especially during the complex breadmaking process, various types of deformation are applied. Beside the application of external forces (e.g., during kneading, sheeting or shaping), the dough is subjected to extension processes as the dough matrix is stretched during the fermentation process. Thereby, the dough matrix, considered as the material located in lamellas around the gas cells, is tangentially extended in two directions and uniaxially compressed in the radial direction while the gas cells increase in size. Different models were applied to estimate extension rates arising during the fermentation. Bloksma (1990) proposed a model consisting of gas-filled cylinders, embedded in the dough matrix phase, undergoing uniaxial extension as the volume of the gas phase increases during fermentation. The extensional strain rates, calculated based on the model of Bloksma (1990) (c.f. Equation (1)), are in the range of 10^−4^–10^−3^ s^−1^ [3].

(1) $$\dot{\varepsilon }=\frac{1}{V_{rel}}\cdot \frac{dV_{rel}}{dt}$$

where V*_rel_* is the relative volume (V_gas + dough matrix phase_/V_dough matrix phase_). In a different approach, van Vliet estimated the biaxial strain of the dough matrix phase around the gas cells [4]. Assuming a constant gas cell number with increasing size over time, the biaxial strain rate is then defined by Equation (2).

(2) $$\dot{\varepsilon }=\frac{dV_{rel}}{3\left(V_{rel}-1\right)dt}$$

The extensional strains found with this approach are comparable to Bloksma’s approach and range from 1.3 × 10^−4^ to 1.1 × 10^−3^ s^−1^, decreasing with increasing fermentation time as the changes in the relative volume decrease [4]. A more practical approach was conducted by Weegels et al. (2016), who traced moving starch granules under the microscope, which were accelerated due to the coalescence of gas cells. Using this approach, maximum extension rates of 10^1^ to 10^2^ s^−1^ were found for proofing dough during the merging process of two gas cells [5]. The presented mathematical models represent an appropriated assumption regarding the overall fermentation process. Thus, as the merging process is part of the destabilisation processes of the dough matrix occurring during the fermentation process, it is necessary to characterise the rheological behaviour of the dough matrix for a broad range of extension rates.

Resisting these biaxial extension forces, the dough matrix is characterised by a high gas retention capacity. This capability is strongly correlated to gluten functionality and its strain hardening characteristics, preventing physical instabilities as coalescence of gas cells and disproportionation. Especially strain hardening behaviour, a phenomenon known in polymer science which can also be observed in dough, requires elongational deformation to be quantified. Undergoing increasing extensional strain, an overproportioned rise in stress is detected at constant strain rates with this phenomenon [4,6].

During the fermentation step, CO_2_ and many secondary metabolites are generated by yeast cells and released into the dough matrix phase due to common metabolic activity, or, in case of osmotic stress, to maintain the internal redox balance [7]. An often-underestimated effect is the impact of secondary metabolites on the rheological properties of the dough matrix, clearly determining the manufacturing properties and end product characteristics [7,8,9,10,11,12,13]. Thus, the aim of the current study is to characterise the rheological behaviour of the fermenting dough matrix for relevant deformation types and strain rates occurring during the breadmaking process. As the behaviour of yeasted dough during the breadmaking procedure is dependent on the exerting load of the current process step and the fermentation-time-dependent metabolite concentration (e.g., CO_2_, acids and ethanol) released into the dough matrix, it is important to characterise dough’s rheological behaviour for deformation types and loads relevant for the breadmaking process. These insights are used to integrate the knowledge of the effects of different yeast metabolites on the rheological behaviour of the dough matrix for the evaluation of strain hardening of fermenting dough at different strain rates. It is hypothesised, that the fermentation process can be considered as an accumulation of metabolites in the dough matrix and the rheological changes can therefore be described by the sum of the impact of each individual metabolite on the dough microstructure. This altered functionality in terms of rheological behaviour is assumed to result from the modified dough microstructure. Therefore, a standard wheat dough, a yeasted dough and a wheat dough spiked with metabolites in yeasted dough-like concentrations, were characterised using shear and elongational rheometry. As elongational deformation occurs during most of the dough processing steps, extensional rheometry provides promising knowledge on practice-related dough behaviour [14], exerting the same load as e.g., fermentation or baking. Lubricated squeezing flow, quantifying the biaxial extensional viscosity, is therefore used to analyse the behaviour of wheat dough and fermented wheat dough at various strain rates. As the extensional strain rates occurring during the breadmaking process were shown to vary within several magnitudes, strain hardening properties were evaluated for a slow and high extension rate within the limits of the applied method. In addition to the rheological characterisation, quantitative microstructural analysis has been shown to be a powerful tool in predicting dough rheology [15]. Protein network analysis (PNA) was thus used to obtain insights into the relation between the rheological behaviour and the microstructural conformation. The obtained knowledge can help to achieve a better understanding of the influence of individual yeast metabolites on the process behaviour of dough during the breadmaking process and support for knowledge-based choice of fermentation parameters beneficiating desirable dough properties.

## 2. Materials and Methods

### 2.1. Wheat Dough Composition

German commercial wheat flour Type 550 with 13.34 ± 0.11 g moisture per 100 g flour (AACCi 44-01), a protein content of 11.26 ± 0.10 g per 100 g dry flour (AACCi 46-16, N × 5.7), 0.55 ± 0.05 g ash per 100 g dry flour (ICC 104/1) and 30.5 ± 0.1 g wet gluten per 100 g flour was used in this study. Dough resistance and water absorption were measured in a Z-kneader doughLAB (Perten Instruments AB, Hägersten, Sewden) according to AACCi 54–70.01 to determine the required kneading time.

### 2.2. Fundamental Shear Rheology

For the shear rheological measurements, 50 g flour (corrected to 14.00% moisture), 3 g white sugar/100 g flour (EC category II quality, Bäko, Nürnberg, Germany) and demineralised water were kneaded for 120 s at 63 rpm using a Z-kneader, equipped with a 50 g bowl. Yeasted doughs were prepared with fresh compressed yeast (*S. cerevisiae*, F.X. Wieninger GmbH, Passau, Germany) at levels of 1 and 2 g yeast/100 g flour. Sugar was added in order to enable unrepressed fermentation by sufficient fermentable sugars. To recreate the level of different metabolites in dough, ethanol, succinic acid and CO_2_ were incorporated in yeast-free doughs in amounts as found by [16]. Respectively 5, 10, 15 or 30 mmol/100 g flour Ethanol (absolute, VWR Chemicals, Darmstadt, Germany) or 0.2, 0.3, 0.4 or 0.5 mmol/100 g flour succinic acid (high purity grade, VWR Chemicals, Darmstadt, Germany) were added, representing the amounts produced by 2 g yeast/100 g flour in dough after 0.5, 1, 2 and 3 h fermentation. To simulate the amount of gaseous CO_2_ released during fermentation, different concentrations of a mixture of Glucono-delta-lactone (Alfa Aesar GmbH & Co KG, Karlsruhe, Germany) and NaHCO_3_ (Solvay GmbH, Hannover, Germany) were used as raising agent. The amount of NaHCO_3_ needed for neutralisation was calculated according to Verheyen et al. (2016) [13]. To adjust the incorporated amount of CO_2_, the volumes of chemically and biologically leavened doughs were compared in graduated cylinders in order to reach the same volume expansion.

After kneading, 7.5 g dough were moulded to sphere form and stored under closed atmosphere (to prevent dehydration) in a drying chamber at 30 °C for periods of 0.5, 1, 2 and 3 h until shear rheological measurements were performed. To standardise the extent of endogenous enzymatic activities and dough relaxation processes, the resting time for metabolite doughs was set to 0.5 h for all metabolite concentrations. Shear rheological measurements were performed using an AR-G2 rheometer (TA instruments, New Castle, DE, USA), equipped with parallel cross-hatched plates (40 mm diameter) at a constant gap of 2 mm. The temperature was constantly controlled to 20 °C by a smart swap peletier plate temperature system. Dough samples were placed centred on the lower plate and excess dough was removed after the gap was set. The cut surface was coated with paraffin oil to prevent dehydration. Subsequently, the sample was allowed to relax for an equilibration time of 10 min before a frequency sweep was performed in a range from 0.1 to 10 Hz at a constant deformation of 0.1%. The obtained complex module data (G*) were fitted to the power law equation (c.f. Equation (3)).

(3) $$G^{*}\left(\omega \right)=A_{f}\omega^{1/z}$$

where ω is the angular frequency (s^−1^), $A_{f}$ refers to the network strength (Pa s^1/z^) and z to the network connectivity (−) [17].

### 2.3. Elongation Properties by Lubricated Squeezing Flow

Due to the higher amount of dough needed for LSF measurements, the dough was prepared in a spiral kneader (KM-25, SINMAG Bakery Equipment, Zuienkerke, Belgium). 2000 g flour (corrected to 14.00% moisture), 3 g white sugar/100 g flour (EC category II quality, Sweet family, Nordzucker AG, Braunschweig, Germany) and tap water were pre-mixed for 30 s and consequently kneaded at 280 rpm for 360 s. For the preparation of yeasted doughs, fresh compressed yeast (*S. cerevisiae*, Uniferm GmbH & Co., Werne, Germany) at levels of 1 and 2 g yeast/100 g flour was added. The dough was sheeted to a final height of 20 mm and cut to cylindrical pieces using lubricated cylindrical cutters (45 mm diameter). The moulded samples were placed into a fermentation chamber (28 °C, 71% rh). The dough samples of the reference and yeast doughs were measured after 0, 4, 7, 10, 30, 60, 120 and 180 min. The metabolite doughs were analysed after 30 min. The measurement was performed with a self-constructed rig for a texture analyser (TA.XT.Plus, Stable Microsystems, Godalming, UK) equipped with a 50 kg load cell based on the experimental procedure described by [18]. The sample was placed between two lubricated perspex plates of 45 mm diameter. The cylindrical sample had the same initial diameter as the upper and lower plate of the experimental setup and was subsequently compressed at displacement speeds of 0.1, 1, 2, 5 and 10 mm/s. The samples were compressed from an initial height of 20 mm to 2 mm, representing a final deformation of 90%. The biaxial strain $\varepsilon_{b}$ and biaxial strain rate $\dot{\varepsilon_{b}}$ were calculated according to equations 4 and 5 [18],

(4) $$\varepsilon_{b}=\frac{1}{2}\ln\frac{h_{t}}{h_{0}}$$

(5) $$\dot{\varepsilon_{b}}=-\frac{\dot{h}}{2h_{t}}=-\frac{v}{2h_{t}}$$

where $h_{t}$ and $h_{0}$ are the thickness at time t and the initial sample thickness and v is the compression speed. The apparent biaxial viscosity was calculated according to Equation (6) [1],

(6) $$\eta_{b}^{*}=-\frac{F_{t}}{\pi r_{p}^{2}\dot{\varepsilon_{b}}}$$

where $F_{t}$ is the force recorded by the load cell at time t and $r_{p}$ is the radius of the plates. The strain hardening index (SHI) was calculated as proposed by [1,19]. Therefore, the stress σ was calculated according to Equation (7),

(7) $$\sigma =\frac{F}{A}$$

with A being defined as the plate area. Stress values for given deformations (0.3, 0.4, 0.5, 0.6, 0.7, 0.8, 0.9, 1.0) were extracted for each measurement at the five compression speeds and were plotted against the biaxial strain rate on a double logarithmic scale. The data for constant deformation was fitted applying a linear model. Using this regression models, stress values were calculated for given values of $\dot{\varepsilon_{b}}$ (0.001, 1.00) and plotted against the deformation on a logarithmic scale. The slope of the linear fit of the plotted data is defined as the SHI, expressed by Equation (8) [1]:

(8) $$SHI={\left(\frac{\delta \ln\left(\sigma \right)}{\delta \varepsilon_{b}}\right)}_{{\dot{\varepsilon }}_{b}=const.}$$

### 2.4. Microstructural Analysis

The protein microstructure was visualised using a confocal laser scanning microscope (eclipse Ti-U inverted microscope with an e-C1 plus confocal system, Nikon GmbH, Düsseldorf, Germany) with a Plan Apo VC 60 ×/1.40 oil objective. The dough was prepared according to the procedure described in Section 2.2 and stained according to the bulk water technique described by [20] using Rhodamine B (0.01 g/100 mL water, Sigma–Aldrich GmbH, Munich, Germany). Proteins were recorded in fluorescence micrographs ($\lambda_{ex}$ = 543 nm, $\lambda_{em}$ = 590/50 nm) with a 1024 × 1024 pixel resolution.

Image processing was performed using the method protein network analysis (PNA) as proposed by [21]. The software AngioTool64 version 0.6a (National Cancer Institute, National Institute of Health, MD, USA, [22]) was applied and the following morphological attributes were chosen for further discussion: Branching rate (number of junctions/protein area), end-point rate (number of end points/protein area), average protein length and width (length and width of a continuous protein strand) and lacunarity (a measure for gaps and irregularities).

### 2.5. Statistical Analysis

All measurements were performed in triplicates. The stated standard deviation accounts for the deviation between triplicates and, in case of modelling, the standard error of the estimates. The propagation of uncertainties was used according to Equation (9) to calculate the absolute error STD [23].

(9) $$ST=\sqrt{{\left(\frac{\delta f}{\delta x}\right)}^{2}STD_{x}{}^{2}+{\left(\frac{\delta f}{\delta y}\right)}^{2}STD_{y}{}^{2}+{\left(\frac{\delta f}{\delta z}\right)}^{2}STD_{z}{}^{2}}$$

where $STD_{x},\;STD_{y},\;STD_{z}$ is the standard deviation of each measurand and $\left(\frac{\delta f}{\delta x}\right)$ is the partial derivation of the function according to each measurand. Mathematical and statistical evaluation was performed using Matlab (R2018a, MathWorks Inc., Natick, MA, USA) and Origin (2018b, OriginLab Corporation, Northampton, MA, USA). Kruskal–Wallis test as a non-parametric test followed by Dunn’s Test as a post-hoc test were used to detect significant differences between groups on a significance level of α = 0.05.

## 3. Results

### 3.1. Network Characterisation under Small Deformation

A frequency sweep at low deformation, within the linear-viscoelastic region (i.e., non-destructive/-structure invasive) was used to quantify the strength of short-range interactions as they occur among starch–starch and starch–gluten polymers [2]. The strength of the short-range interactions was quantified in terms of network strength A_f_ and network connectivity z. Those coefficients were obtained using the power law equation to fit the frequency dependency of the complex shear modulus G*.

**Figure 1:**
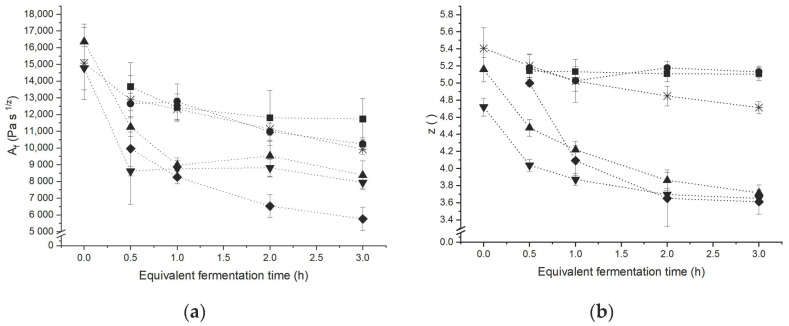
Effect of different yeast levels and yeast metabolites on power law constants during 3 h fermentation at 30 °C. (**a**) Network strength A_f_. (**b**) Network connectivity z. (□) Reference without additives, (▲) 1 g yeast/100 g flour, (▼) 2 g yeast/100 g flour, (♦) CO_2_, (■) succinic acid, and (●) ethanol. The term equivalent fermentation time does not represent a real time for metabolite spiked doughs as those doughs contain the same amount of metabolites as [16] quantified in fermenting doughs (2 g yeast/100 g flour) at the marked time points, but rested for only 0.5 h after kneading. (n = 3, $\bar{X}$ ± STD).

A standard wheat dough serves as a reference point since this corresponds to the untreated case. As shown in Figure 1, the rheological behaviour of standard wheat dough is shown to be dominated by relaxation processes of the gluten structure as a time-dependent weakening of the dough structure occurred during dough resting. The network connectivity, a measure for the extend of interactions within the structure, slightly decreased with increasing resting time. On a molecular level, this relaxation behaviour is attributed to the conformational changes occurring as the polymer matrix is attempted to reach a thermodynamically preferred equilibrium. Along those changes, occurring after the termination of energy input throughout the kneading step, the proteins abandon their unfolded and extended conformation and return to a compact structure. Due to this process, the system has a decreasing ability to form intermolecular forces. After the kneading step, the protein strands are in a strongly aligned conformation and are forced to interact among shortrange interactions among the highly extended, densely packed strands. With increasing resting time, the loop-train structure is re-achieved and the high number of forced, low entropic interactions is replaced with fewer, more stable interactions. This was shown to prevail the re-polymerisation process upon the reformation of S-S-bonds, as the network connectivity shows an overall decrease during dough resting. Furthermore, the decreasing consistency was related to conformational changes during the relaxation process in terms of the re-formation of loop regions based on the loop and train model proposed by [24]. Additionally, endogenous enzymatic activity might contribute to the time-dependent behaviour of non-yeasted standard wheat dough [25].

As might be expected, the presence of yeast caused the dough network to turn considerable instable. The network structure appears less stable as the network branching and strength of the interactions are markedly lower. Those effects, comprising the structural degradation of the dough microstructure, were shown to be concentration dependent as the network is shown to be substantially more instable with a higher yeast level. The origin of the above-mentioned structural changes, causing the modified rheological behaviour, is assumed to be caused by the rupture of protein strands inside the gas cell-surrounding lamella due to the mechanical forces exerted by expanding gas cells [26]. To verify this assumption, different concentrations of yeast metabolites found during the fermentation process have been used in spiking experiments to reconstruct doughs in order to reformulate a fermenting dough. To reduce the complexity and enhance the validity, only one metabolite at once was used in the metabolite doughs.

Initiated by the presence of gaseous CO_2_ in fermenting dough-like amounts, the frequency dependence was shown to be increased and the consistency was lowered like in yeasted dough as indicated in Figure 1. These findings are in agreement with Chin et al. (2005) who found the presence of CO_2_ to decrease the load capacity of dough, especially the failure stress was shown to develop indirect proportional to the gas void fraction [27]. As the amount of CO_2_ produced over time increases and gas cells increase in size, the increasing gas void fraction is leading to a growing spatial demand. Thus, the interactions of dough polymers are sterically hindered by the presence of gaseous CO_2_ in the raising dough matrix. Beside gaseous CO_2_ as a main metabolite, it appeared that the presence of ethanol and organic acids caused only minor changes. Ethanol was found to affect the dough matrix behaviour only for high concentrations. This confirms the results of [9,10], who found ethanol to influence the dough structure at concentrations above 2 *v*/*v*%. Such an ethanol concentration would only be reached after 3 h at a yeast level of 5.3 g/100 g flour [16], which represents a rather high concentration for the production of baked goods. This indicates that this metabolite has no weakening influence on dough structure during the early fermentation state. The effects of this metabolite can only be seen in the latter fermentation state, where ethanol causes a slight increase in the number of interactions (z ↑). At the same time, the strength of the system was shown to be decreased (A_f_ ↓), which probably reflects the dilution effect caused by the increasing amount of liquid present in the system. According to the Osborne fractionation, ethanol acts as solvent for gliadins. Therefore, it is expected, that this gluten fraction shows a swelling behaviour which causes the reduction of the reinforcing effect of gliadins on the glutenin network as previously reported by [28]. For succinic acid, a re-enforcing effect on the dough matrix functionality has been observed. Succinic acid enabled a higher network connectivity and strength (z and A_f_ ↑). This matched the findings of Wehrle (1997), who found lactic acid and acetic acid to cause a more elastic and firmer dough behaviour [29]. Further, Jayaram (2014) reported succinic acid to stiffen the dough structure, accompanied by a decreasing dough extensibility. The authors attributed this behaviour to unfolding and swelling of the gluten proteins in presence of succinic acid. As the network connectivity z was shown to be markedly increased in presence of succinic acid, these conformational changes are considered to increase the number and strength of possible interactions due to the greater accessibility upon the unfolded and elongated protein conformation. This indicates that small amounts of acid, as present on fermented yeasted dough seem to strengthen the dough structure and increase the stability of the system.

The results of this section indicate that CO_2_ has the greatest functional influence on the rheological behaviour of dough under small deformation. The destabilising effect of gas was shown to predominate over the reinforcing effect of acid in fermented dough.

### 3.2. Extensional Viscosity of Yeasted Wheat Dough

**Figure 2:**
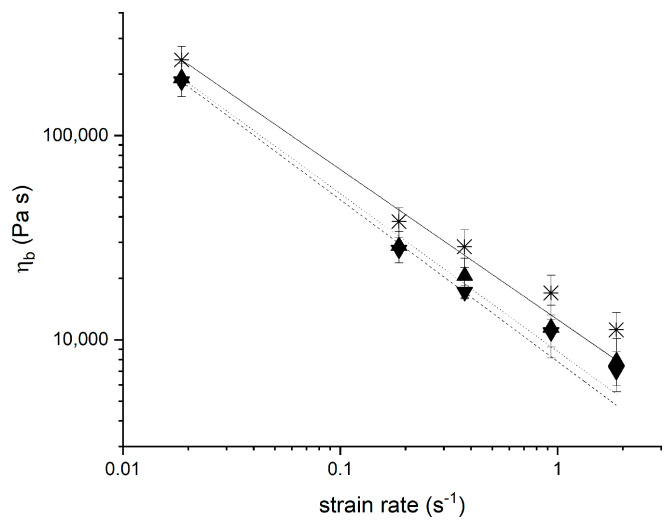
Extensional viscosity measured at ε_b_ = 1.00 after 3 h fermentation at 30 °C presented as flow curve. Data were fitted according to Turbin-Orger et al. (2015) using a power law model ($\eta_{b}\;\left(\dot{\varepsilon }\right)=K\cdot {\dot{\varepsilon }}^{\left(n-1\right)}$). (―□―) Reference without additives, (··▲··) 1 g yeast/100 g flour, (--▼--) 2 g yeast/100 g flour. (n = 3, $\bar{X}$ ± STD).

**Figure 3:**
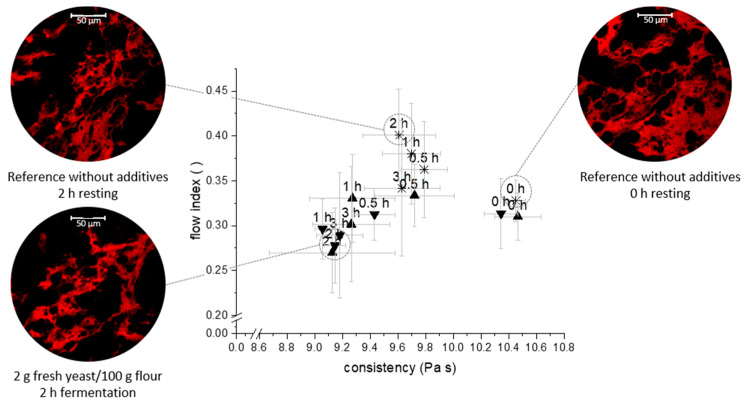
Time-dependent effect of fermentation on the flow index n and consistency index K for $\varepsilon_{b}$ = 1.00. Selected micrographs (protein stained with Rhodamine B (red)) illustrate the corresponding dough microstructure. (□) Reference without additives, (▲) 1 g yeast/100 g flour, (▼) 2 g yeast/100 g flour. The CLSM images are shown at a scale of Ø 225 µm. (n = 3, $\bar{X}$ ± STD).

Beside the characterisation in the linear viscoelastic shear flow regime, an extensional flow technique was applied to analyse the extensional viscosity of the system (Figure 2). Dough as a polymeric system with gluten as three-dimensional entangled macromolecule is characterised by a high extensional viscosity [30]. Since the gained flow curves show a linear relation within extensional viscosity and extension rate (R^2^_adj_ = 0.99), a power law model (c.f. equation 10) can be employed to analyse the flow behaviour

(10) $$\eta_{b}\;\left(\dot{\varepsilon }\right)=K\cdot {\dot{\varepsilon }}^{\left(n-1\right)}$$

where K (Pa s^n^) is the consistency index and n (-) is the flow index [31]. Seeing the flow index being lower than 1, the general dough elongational behaviour within the considered region can be described as extension thinning (Figure 3). Such a flow behaviour is well known for polymer solutions and can be contributed to orientation of the molecules along the flow direction. The spatial reorientation and partial collapse of the dough microstructure causes a decreasing stress with further extension as long as no stretching of the polymers occurs [32,33,34]. By extending the dough matrix, secondary bonds are broken and protein strands are able to slip past each over [35]. During further extension, rupture of gluten strands and the alignment and separation of starch granules within the gluten matrix contribute to the extension thinning flow behaviour of a standard wheat dough [36].

The presence of yeast was shown to reduce the extensional viscosity. The viscosity drop is even more pronounced with increasing fermentation time and a higher yeast level. Thus, the fermentation activity of yeast is considered to cause the system to be less connected or entangled. The re-orientation of the system according to the deformation force is promoted, since the system has a higher mobility due to shifted polymeric structure. This alteration is presumably attributable to the occurrence of shorter chained polymers upon the rupture of gluten strands due to the fermenting activity, causing the polymeric structure to be less branched and entangled [34].

### 3.3. Linkage between Extensional Rheological Properties and Protein Network Configuration

In order to validate the hypothesised correlation between the flow behaviour and the microstructure of the polymeric system, quantitative protein network analysis has been performed to address the microstructure of standard wheat dough and yeasted dough. In order to distinguish between yeast- and time-dependent effects, the effects of resting time and fermentation were evaluated (Table 1).

Directly after kneading, the dough microstructure appeared as a strongly interconnected network. Shear, elongational and rotational deformation forces during the kneading caused the formation of a highly aligned protein microstructure [37]. Especially the extension of strands during kneading forces the formation of additional shortrange interactions (e.g., hydrogen bonds) due to the more extended and unfolded protein configuration. Thus, the network appears dense and comprises wide and planar strands. Since the proteins were not allowed to regain their thermodynamic equilibrium conformation, the system appears in a strongly connected arrangement directly after the kneading process. After sufficient resting time (cf. standard wheat dough without additives, 2 h resting), the conformational changes and rearrangement of protein strands cause a decreasing vessel length (−26.6%) due to the contraction of the protein threads. Further, the connectivity of the network was shown to be changed by relaxation processes. This relaxation processes include the continuous hydration of the dough system during resting and are emphasised to enable a higher molecular mobility and the formation of new interactions, supporting the regaining of a low entropy for the system. In contrast, the appearance of the system changed in presence of yeast. The most marked change was the reduced density and grown spatial separation of protein strands. During the fermentation step, the yeast metabolite CO_2_ is known to dissolve from the liquid dough phase, leading to an increased gas void fraction. Thus, steric separation of protein structures and hindered interactions along protein strands reduce the width of protein threads due to compressive stress. Beside the compressive stress, the fermentation processes were shown to cause elongational strain leading to more pronounced restructuring processes and additional damage. In view of the reported data, this effect is represented by a significant decrease in number of junctions and an increase in end points. The observed changes can be summarised according to the classification scheme for gluten networks of [15]. It appears that yeast encourages the formation of a “particulate, dense network” where the proteins form clustered agglomerates and are densely arranged.

As abovementioned, the stress thinning behaviour suggest power law behaviour. The results of power law modelling are presented in Figure 3 in terms of flow and consistency index. The presented parameters underline the previously discussed changes. In general, the standard wheat dough without additives or yeast appears to decrease in consistency with increasing resting time, accompanied by an increase in crosslinks. This is expected to be caused by the rearrangement of the gluten network during the resting period, since the entropy changes during resting are known to increase the loop/train ratio. Contrary to that, the rheological behaviour of yeasted dough is marked by a decreasing consistency combined with decreasing flow index with increasing fermentation time, which has been associated to a higher mobility of biopolymers [38]. It is likely that the decreasing number of crosslinks provokes this altered flow behaviour. The drop is ascribed to the rupture of gluten strands due to the extensional load exerted from the growing steric demand of gas cells. Further, ethanol has been shown to reduce the strength of interactions within the system and could therefore contribute to the weakening of the system.

It is apparent that the flow behaviour is strongly linked to the functionality of the dough microstructure, as previously reported by other authors [15,39]. In the previous section, the impact of resting and fermentation processes on the dough microstructure and flow behaviour has been reported. As presented in Figure 3, the results indicate those processes to influence the functionality of the dough matrix considerable differently. During resting, relaxation processes occur, causing a stress reduction and enabling a higher deformability compared to unrested dough. This can be interpreted from the decaying values of K during the resting period of dough. The increase in the flow index n can be attributed to repolymerisation processes upon the reformation of S-S-bonds, providing a higher internal stability within the protein network than hydrogen bonds. In contrast to that, the fermentation process counteracts the formation of a stable, relaxed network arrangement. Additionally, the growing amount of gas impairs the protein strands (lacunarity ↑) and causes strand rupture (length ↓). Therefore, the amount and strength of interactions along the proteins decrease (branching rate ↓). The resulting fragments are more accessible to deformation upon mechanical exposure and the system has a lower yield strength.

### 3.4. Strain Hardening Behaviour of Yeasted Wheat Dough

The fermentation process has been shown previously to clearly impact the microstructure and functionality of the dough matrix. One of the most important functions of the dough matrix is the ability to retain and stabilise gas cells. As mentioned in the introduction, the gas holding capacity of dough is strongly correlated to the strain hardening phenomenon, which occurs in polymeric systems consequent to the exertion of extensional strain. To quantify the ability of a polymeric system to establish strain hardening behaviour, Jansen et al. and van Vliet et al. introduced the strain hardening index [4,40]. This was later adapted by Rouille et al., whose definition was applied for the present calculations [1]. In general, the strain hardening index (SHI, cf. equation 8) is defined as derivation of ln(σ) on $\varepsilon_{b}$ at constant $\dot{\varepsilon_{b}}$. According to its mathematical definition, strain hardening occurs if $\frac{dln\left(\sigma \right)}{dln\left(\varepsilon \right)}>1$ [4]. Regarding the underlying mechanism of this phenomenon, it is believed to be triggered by the formation of multi-branch structures in the gluten network. Those arrangements decrease the mobility of the system and give rise to an increased stress level during the deformation [36]. On a molecular scale, strain hardening was shown to result in extended HMW subunits, a higher β-sheet content and lower levels of turns [41]. Those changes were shown to be reversable but affected the rheological properties within the first minutes after extension. Further, an incomplete relaxation is assumed, as the extend of conformational changes increases with repeated deformation activity. The polymeric dough system is therefore assumed to serve from a stress memory. This memory behaviour is based on the non-reversible changes occurring in the protein network during the fermentation process. In order to evaluate the effects of yeast fermentation on the strain hardening behaviour of the dough matrix, the time-dependent SHIs were analysed for a standard wheat dough, yeasted doughs and metabolite-spiked doughs at two different strain rates. The chosen strain rates represent the lower and upper measuring limit of the method used and were chosen to reflect slow extension processes (e.g., fermentation) and processes at a high extension rate (e.g., oven rise during baking and shaping).

**Figure 4:**
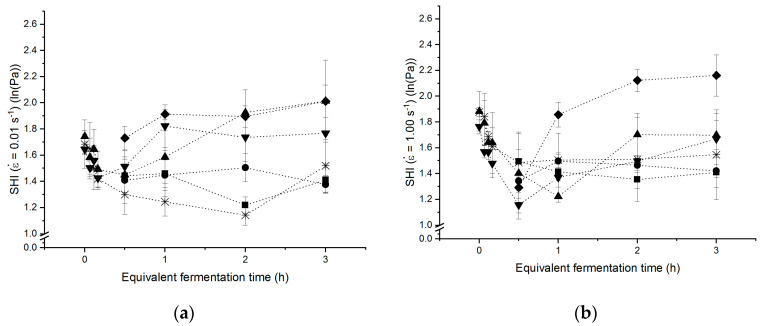
Strain hardening behaviour of yeasted dough with different yeast concentrations and yeast metabolites during 3 h fermentation at 30 °C. (**a**) Strain hardening index for an elongation rate of $\dot{\varepsilon_{b}}$= 0.01 s^−^^1^. (**b**) Strain hardening index for an elongation rate of $\dot{\varepsilon_{b}}$ = 1.00 s^−^^1^. (**□**) Reference without additives, (**▲**) 1 g yeast/100 g flour, (**▼**) 2 g yeast/100 g flour, (**♦**) CO_2_, (**■**) succinic acid, (**●**) ethanol. (n = 3, $\bar{X}$ ± STD).

The data in Figure 4 illustrates that the strain hardening behaviour of a standard wheat dough is considerably affected by relaxation processes occurring during the resting of dough consecutive to the kneading process. Regardless the extension rate applied, particularly the first minutes after kneading led to a less dominant strain hardening behaviour. This decay is probably caused by the increase in the loop/train ratio during dough resting [24]. On the whole, the SHI was moderately higher upon the extension at high strain rates, compared to the deformation at a low extension rate. This discrepancy is initiated by the contrasting mechanisms arising from the different extension rates. During the extensional deformation at low extension rates, a breakage of secondary bonds causes the slippage of strands past each other, while at the high extension rate, the network breaks into subunits which might entangle with each other and therefore show a higher resistance to extension. Further, separation and re-alignment of starch granules, acting as fillers within the extended gluten matrix, give rise to friction between starch granules originating in a higher resistance to extension.

It appears that the presence of acid or ethanol, representing the most common yeast metabolites, is not related to a clear functional influence on the strain hardening properties as the SHI obtained for those doughs do not differ significantly from the standard dough. Both metabolites showed no clear concentration dependent effect within the test range. Ethanol initiated a slightly enhanced strain hardening behaviour at a low strain rate. It can be assumed, that this behaviour results from lack of gliadins within the gluten network as gliadins are generally considered to contribute the viscose share to the viscoelastic dough properties. By lacking this fraction due to the increased solubilisation of this fraction within the liquid dough phase, the extension might be hindered as the elasticity of the system increases. Especially at higher concentrations of this metabolite and faster extension rates, the reinforcement of the system is limited, as the number of available binding sites is reduced. Succinic acid, representing the impact of organic acids on the dough matrix during fermentation, does not cause a clear overall trend. In line with the previous results, the protein conformation is assumed to be more open but simultaneously more connected as well. These contradictive processes contribute to the minor impact of this metabolite on the strain hardening functionality of the dough matrix during large deformation processes.

Unlike the previous discussed metabolites, yeasted and chemically leavened dough were substantially affecting the strain hardening behaviour. As both doughs are affected in the same manner, it appears that gaseous CO_2_ is responsible for this effect. The presence of gas cells emphasises the SHI to increase considerably. It is assumed that this increase is due to gas serving as preload on the gluten network. Upon this initial load, the gluten strands are already in an extended conformation. Consequently, additional external elongational deformation cannot be tolerated to the same extend as in unleavened dough and, thus strain hardening occurs to a higher extend. As reported by Belton (2005), repeated extension of gluten leads to an increase of β-sheets. It is likely that these changes within the protein structure are promoted during the pre-extension of the system while fermentation and contribute to the accelerated strain hardening behaviour during further extension [41]. It is further hypothesised that starch granules are rearranged within the gluten matrix due to the increasing gas void fraction. Starch is therefore compressed and forms compartments within the gluten strands and gas cells. Thus, it is no longer homogeneously distributed and causes higher local stress due to higher stiffness. The comparison within slow and fast extension rates reveals a more prominent strain hardening behaviour at a higher strain rate. This is in good agreement with the trend observed for unleavened standard wheat dough and traced back to the same underlying mechanism. As suggested earlier, slippage and intermediate stabilisation along protein strand occur at low strain rates, whereas structural break down is initiated at high deformation rates. As the presented data suggests a more pronounced strain hardening behaviour occurs during fast extension processes. The results indicate therefore that yeasted dough is a self-enforcing system, as (i) gas retention capacity requires strain hardening, and (ii) strain hardening is enhanced through fast gas formation (i.e., high strain rates). This observation is in good agreement with the result of Verheyen et al. [13]. The authors measured the gas holding capacity in dependency of the yeast level for fermenting dough by several macroscopic approaches, and found an enhanced retained CO_2_ volume with higher CO_2_ formation kinetics. Using a spiking-approach in combination with micro- and macrostructural analysis, it was possible to trace the mechanism behind this self-enforcement of yeasted dough systems back to the release of gaseous CO_2_ into the dough matrix in the present study.

## 4. Discussion

**Figure 5:**
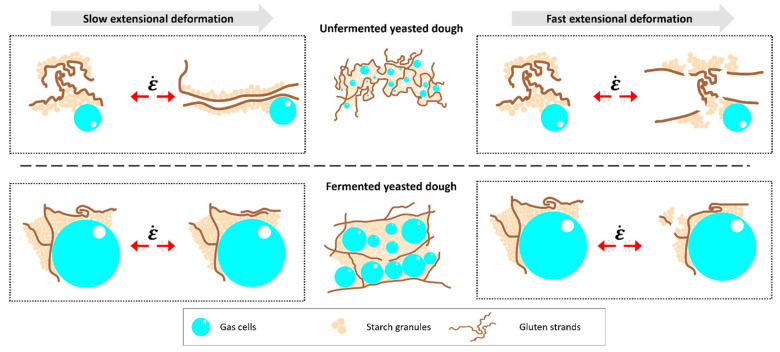
Schematic illustration presenting the hypothesised network model during extension processes for unfermented and fermented dough, describing the observed effects from Figure 4. The scheme illustrates the possible pathways for slow (left) and fast (right) extension rates ($\dot{\varepsilon }$) of unfermented (top) and fermented (bottom) dough matrix. The underlying information on the gas void fraction present in unfermented and fermented dough are 0.1 and 0.75, respectively [42].

Figure 5 proposes a schematic mechanism for extension processes of dough matrices. Two different scenarios are considered in this scheme: Unfermented dough with a gas void fraction of 0.1 and fermented dough with an increased gas void fraction of 0.75 and an increased gas cell size [42]. As stated previously, strain hardening was shown to be strongly dependent on the applied extension rate. In case of a slow extensional deformation, the gluten network can react to the deformation force by the extension of loop regions. Further, the slippage of gluten strands along each other is promoted due to the re-stabilisation ability among shortrange interactions. These interactions occur along gluten strands as well as along starch and gluten and enable an intermediate stabilisation while the extension process is conducted and prevent rupture of strands upon extension. Contrary, at a fast extension rate, intermediate stabilisation of the system is prevented due to the higher ratio of bond breakage to bond reformation. This results in the breakage of gluten strands and break apart of starch clusters. Fermentation was shown to cause a pre-load on the dough matrix. The stress memory of the gluten network is believed to be biased by the fermentation activity, which causes pre-extension and potential rupture of gluten strands due to the growing spatial demand of CO_2_ cells, as stated in Section 3.3. Therefore, interactions within the dough polymers are sterically hindered and the strength of the gluten network is reduced. Similarly, as in the case of the unfermented dough, it is likely that at a low strain rate (Figure 5, left side, bottom), intermediate stabilisation via shortrange interactions among gluten–gluten, gluten–starch and starch–starch, results in a low strain hardening index. As starch is successfully stabilised within the gluten matrix via transitory shortrange interactions, the occurrence of friction within the compressed starch clusters and gluten strands is avoided and starch contributes to the stabilisation of the polymeric system during the extension process. In contrast to that, the potential mechanism for extension at a high strain rate (Figure 5, right side, bottom) is related to a breakdown of the network structure upon extension. The polymeric network fragments, caused by the pre-load of fermentation, remain entangled due to the fast extension process. The entrapped polymers cannot undergo a free slippage process and the resulting thinning process increase the resistance to extension. Further, the stabilisation of the system by intermediate polymer–filler-interactions is hindered. This is due to (i) the displacement of starch granules from the thinning dough matrix lamella around the growing gas cells during the fermentation process and (ii) the compression of starch granules into densely packed clusters, whose formation is promoted by (i). These clusters cause frictional forces between gluten and starch granules, resulting in additional load on the gluten strands. Consequently, higher resistance to extension is promoted and strain hardening is accelerated. Further, breakage of strands is enhanced due to the occurrence of locally high frictional forces between starch clusters and gluten strands. In summary, our results suggest that the strain dependent behaviour is strongly affected by the stress memory of the gluten network. Fermentation causes irreversible changes in the polymeric structure, affecting the strain hardening behaviour of fermented dough system. The generated knowledge provides a fundamental understanding of the influence of yeast fermentation on the behaviour of dough during strain rate dependent extension processes. The obtained insights can be used to provide a base for the choice of fermentation parameters beneficiating desirable dough properties and a better understanding of processability of fermented dough during the breadmaking process.

## 5. Conclusions

The fermentation metabolite CO_2_ had the strongest impact, as expanding gas cells destabilised the dough matrix. Thus, the extent and strength of interactions at all structural levels were reduced due to the growing steric demand of the gas void fraction. Dough incorporating gaseous CO_2_ behaved highly complementary to fermenting dough. The gas void fraction thus mainly governs the properties of yeasted dough. Organic acids and ethanol had only a limited impact, especially during the first initial phase of the fermentation process. Therefore, the acidification and the accumulation of ethanol seem to influence the behaviour of yeasted dough only marginally.

The polymeric and entangled gluten structure, being quantitatively degraded throughout the fermentation process, defined the flow behaviour of fermenting dough. The microstructural destabilisation process was successfully related to the changing rheological behaviour towards a highly mobile polymer system, represented by a lower flow index. Besides the time-dependent changes during fermentation, the dependency on the load type and strain rate determined the rheological properties of fermenting dough. This includes extension-thinning behaviour and a dependency of the strain hardening behaviour on the applied strain rate. The dependency was mainly traced back to the presence of growing gas cells, which accelerated the strain hardening behaviour. This might be due to two reasons: (i) The growing steric demand of gas cells, causing a pre-extension of the gluten strands within the lamella around the gas cells and (ii) may be, as a side effect, starch granules accumulating and causing higher friction on the polymer strands around these fillers. This second effect might occur even more pronounced at a high extension rate. In general, shortrange interactions contributed to the stabilisation of the polymeric system and therefore enabling a free slippage of elongated, disentangled strands at a low extension rate. At a high extension rate, the interference from broken-apart, sterically interacting fragments seems to accelerate the strain hardening behaviour of dough.

## Figures and Tables

**Table 1:** Mean values of PNA results. The dough samples were stained with Rhodamine B using the bulk water technique and analysed by CLSM with 60 × magnification. (n = 3, $\bar{X}$ ± STD).

	Average Protein Vessel Length (µm)	Average Lacunarity (-)	Branching Rate (Junctions/µm^2^)	Endpoint Rate (End Points/µm^2^)	Protein Width (µm)
Standard wheat dough without additives 0 h resting	7217 ± 2586 ^a^	0.35 ± 0.04 ^a^	6.1·10^−5^ ± 0.4·10^−5 a^	1.6·10^−5^ ± 0.2·10^−5 a^	69.53 ± 1.54 ^a^
Standard wheat dough without additives 2 h resting	5298 ± 1307 ^a^	0.35 ± 0.06 ^a^	5.8·10^−5^ ± 0.5·10^−5 a^	2.1·10^−5^ ± 0.3·10^−5 b^	66.07 ± 0.66 ^b^
2 g yeast/100 g flour 2 h fermentation	4503 ± 1511 ^b^	0.41 ± 0.12 ^a^	5.6·10^−5^ ± 0.4·10^−5 b^	2.2·10^−5^ ± 0.4·10^−5 b^	66.95 ± 1.81 ^b^

a,b Mean values ± STD labelled with a different letter in the same column are significantly different according to Dunn’s Test on a significance level of α = 0.05.

## Data Availability

Data is contained within the article.
